# ﻿A new species *Erythrotrichiabohanensis* sp. nov. (Erythropeltales, Rhodophyta) from the coast of China

**DOI:** 10.3897/phytokeys.256.145842

**Published:** 2025-05-29

**Authors:** Bingxin Huang, Yue Chu, Yuan Gao, Yanguang Zhong, Meina Chen, Chang Sun, Lanping Ding

**Affiliations:** 1 College of Life Sciences, Tianjin Normal University, Tianjin 300387, China Tianjin Normal University Tianjin China; 2 Tianjin Key Laboratory of Animal and Plant Resistance, Tianjin 300387, China Tianjin Key Laboratory of Animal and Plant Resistance Tianjin China

**Keywords:** Cryptic species, Erythropeltales, *Erythrotrichia*, new species, taxonomy

## Abstract

*Erythrotrichia* (Erythropeltales, Rhodophyta) is a group of widely distributed marine epiphytic algae. With the advancement of molecular biology in recent years, the classification of this genus has undergone significant changes, revealing several morphologically indistinguishable cryptic species. In this study, we collected species of *Erythrotrichia* from the Bohai Sea coast of China, proposed a new species—*Erythrotrichiabohanensis***sp. nov.** based on laboratory culture, morphological observation and molecular phylogenetic analysis. Phylogenetic analyses, including *rbc*L and SSU gene sequence analyses, demonstrated that *Erythrotrichiabohanensis***sp. nov.** clusters into an independent branch with high Maximum Likelihood (ML) bootstrap values and Bayesian Inference phylogenies (BI) posterior probabilities. The new species is characterized by an unbranched, filamentous thallus and exhibits both asexual and sexual reproduction. The main morphological differences of the new species are primarily reflected in its sexual reproduction process. The carpogonium and spermatium are formed by the expansion and division of vegetative cells, and fertilization occurs outside the thallus. The establishment of this new species underscores the cryptic species diversity within this taxon and implies that additional morphological, molecular, and genetic information is essential for more precise species identification.

## ﻿Introduction

The order Erythropeltales is a monophyletic group that encompasses only one family Erythrotrichiaceae, which is widely distributed globally (Guiry and Guiry 2025). The members in this order exhibit morphological simplicity, which is characterized by the presence of structures such as filaments, crusts, or simple blades (Zuccarello et al. 2010). Additionally, they are uniformly defined by the presence of a central plastid containing a pyrenoid and the absence of pit plugs (Zuccarello et al. 2010; Guiry and Guiry 2025). The genus *Erythrotrichia*, the most species-rich genus within the Erythrotrichiaceae, was established by Agardh in 1883 and now 31 species have been recorded from around the world (Guiry and Guiry 2025). These epiphytes grow on other algae or seagrasses, as uniseriate or multiseriate filaments reaching only a few millimeters in height (Zheng and Li 2009). Each cell contains a single, stellate chloroplast with central pyrenoid (Zuccarello et al. 2010). Asexual reproduction is via monospores (Zheng and Li 2009; Zuccarello et al. 2010). A vegetative cell divides obliquely into two cells of unequal size, with the smaller cell developing into the monosporangium (Zheng and Li 2009). While sexual reproduction has been documented in some species, the sexual cycle remains incomplete (Magne 1990).

In recent years, the field of molecular phylogenetic analysis has catalyzed significant shifts in the taxonomic study of *Erythrotrichia*, challenging traditional classification methods that rely only on morphological characters. Many morphologically similar species exhibited substantial genetic differences (Zuccarello et al. 2011; West et al. 2012). The genus *Erythrotrichia* is now recognized as comprising six distinct clades (Zuccarello et al. 2011; West et al. 2012), a division that separates species previously grouped under a single morphological classification. These insights reveal the presence of multiple cryptic species within *Erythrotrichia*. Although these species are morphologically indistinguishable, they exhibit clear molecular distinctions (Zuccarello et al. 2011). As a result, several new species have been described in recent years, including *E.longistipitata* (West et al. 2012), and *E.johnawestii* (Wen et al. 2023). Despite these advancements, there are still unnamed taxa that require further investigation.

Surveys conducted in the Yellow and Bohai Seas have revealed the presence of species from the genus *Erythrotrichia* with 11 species, given that these identifications primarily relied on morphological character classification (Luan and Luan 2005; Zheng and Li 2009; Ding et al. 2015). In this study, we isolated an epiphytic *Erythrotrichia* sp. from a *Polysiphonia* sp. in laboratory culture that had been collected from the Bohai Sea, and combined molecular data and morphological characters to determine its species status.

## ﻿Material and methods

### ﻿Samples collection, isolation and culturюe

Host specimens of *Polysiphonia* sp. were collected from Dongshan Beach (39°54'N, 119°37'E), Qinhuangdao, Hebei, China in March and May 2023, and were cultured under laboratory conditions. After one month of cultivation, *Erythrotrichia* sp. were observed as epiphytic growths on hosts. Individual *Erythrotrichia* sp. samples were isolated from four hosts with forceps and needles followed by isolation with 200 μL pipettes under a stereo microscope (Phenix XTL-165, Shanghai, China). These hosts were identified as two species, *Polysiphoniasenticulosa* Harvey and *Polysiphoniamorrowii* Harvey. The isolated samples were subsequently cultured separately in salinity 30 sterilized natural seawater at 15 °C, 12.5 μmol photon m^-2^ s^-1^, 12 L:12 D photoperiod in a light incubator (Intelligent Light Incubator GXZ-380B-LED, Ningbo Jiangnan Instruments, China).

### ﻿Morphological identification

The host algae were observed under a stereo microscope (Phenix XTL-165, Shanghai, China) to determine the attachment location and attachment mode. Specimens were observed under a microscope (Phenix PH100, Shanghai, China), and photographs were taken of key identification features. The vegetative and reproductive structures of the alga were compared and analyzed.

### ﻿DNA extraction, PCR amplification and sequencing

DNA was extracted using TIANGEN Rapid DNA Extraction Detection Kit KG203 (TIANGEN Biochemical Technology, Beijing, China). DNA concentration and purity were detected using NanoDrop one (ThermoFisher Scientific, Shanghai, China).

PCR amplification of *rbc*L and SSU fragments was performed. PCR reaction system was 20 μL, 2×Det PCR MasterMix 10 μL, 0.5 μL each of forward and reverse primers, 1 μL of DNA template, and 8 μL of ddH_2_O. For small subunit ribosomal RNA (SSU), primers were selected from G04–J04 (Saunders and Kraft 1994). The PCR conditions were: pre-denaturation at 96 °C for 30 s, followed by 35 cycles of denaturation at 94 °C for 30 s, annealing at 50 °C for 1 min, extension at 72 °C for 2 min, and a final extension at 72 °C for 10 min. For ribulose-1,5-bisphosphate carboxylase/oxygenase large subunit (*rbc*L), primers were selected from Comp1(A)–Comp2(A) (Rintoul et al. 1999) and F57–R753 (Freshwater and Rueness 1994). The PCR conditions were: pre-denaturation at 95 °C for 2 min, followed by 35 cycles of denaturation at 93 °C for 1 min, annealing at 50 °C for 1 min, extension at 72 °C for 4 min, and a final extension at 72 °C for 2 min.

The PCR products were detected by 1% agarose gel electrophoresis and then sent to commercial sequencing; the sequencing results were checked using Chromas 2.6.6.

### ﻿Phylogenetic analysis

A total of 86 DNA sequences were utilized in our analyses, with specific sample details provided in Suppl. material 1, including four SSU sequences and three *rbc*L sequences newly generated in this study. Multiple sequence alignment and phylogenetic tree construction were performed using PhyloSuite v1.2.3 (Zhang et al. 2020; Xiang et al. 2023). Sequences were aligned with MAFFT v7.505 (Katoh and Standley 2013). Maximum likelihood phylogenies (ML) were inferred using IQ-TREE v2.2.0 (Nguyen et al. 2015) under the TVMe+G4 model for *rbc*L and the K2P+I model for SSU, both selected by IQ-TREE. These analyses were performed for 1000 standard bootstraps. Bayesian Inference phylogenies (BI) were inferred using MrBayes v3.2.7a (Ronquist et al. 2012) under SYM+G model for *rbc*L (1 parallel runs, 2000000 generations), and K80+I model for SSU (1 parallel runs, 2000000 generations), in which the initial 25% of sampled data were discarded as burn-in. Phylogenetic tree visualization and landscaping were performed using tvBOT (Xie et al. 2023).

## ﻿Result

### ﻿Phylogenetic analysis

**Figure 1.**
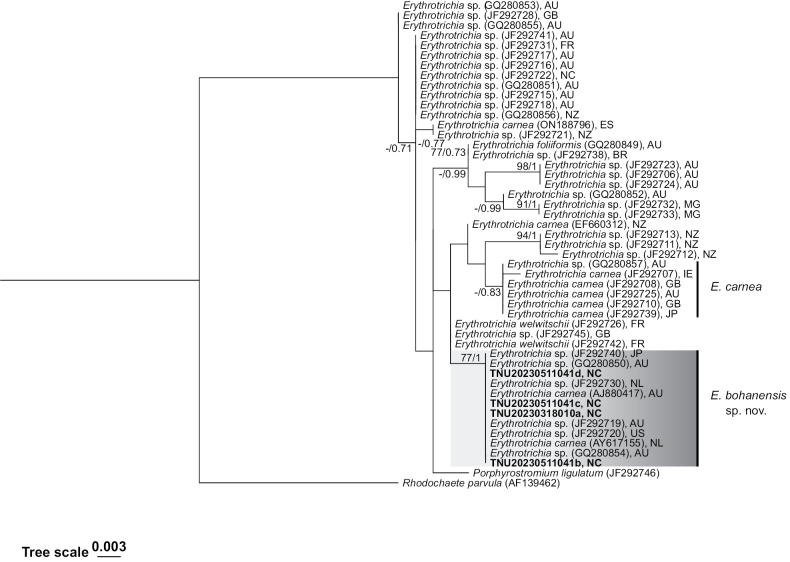
Phylogenetic tree constructed based on SSU sequence fragments. The values on the branch represent the ML bootstrap values (left) and Bayesian posterior probabilities (right) ˳“-” indicates ML bootstrap values < 70 or BI posterior probabilities < 0.7. Bold font indicates samples from this study.

**Figure 2.**
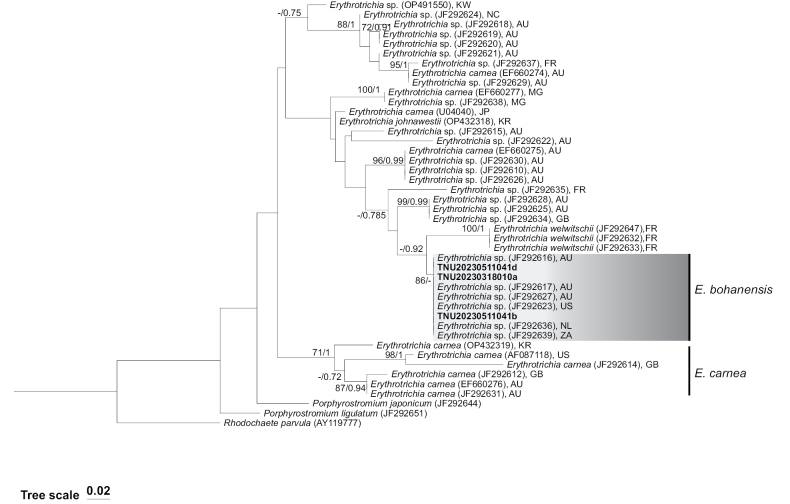
Phylogenetic tree constructed based on *rbc*L sequence fragments. The values on the branch represent the ML bootstrap values (left) and Bayesian posterior probabilities (right) ˳“-” indicates ML bootstrap values < 70 or BI posterior probabilities < 0.7. Bold font indicates samples from this study.

ML and BI analyses based on SSU gene sequences produced the same tree topology, as shown in Fig. 1. The four samples in this study (TNU20230318010a, TNU20230511041b, TNU20230511041c, and TNU20230511041d) were compared with an unnamed *Erythrotrichia* species from Japan, Australia, and the Netherlands, showing high support (ML bootstrap values = 77, BI posterior probabilities = 1). In addition, ML and BI analyses based on *rbc*L gene sequences yielded the same tree topology, as shown in Fig. 2. The three samples in this study (TNU20230318010a, TNU20230511041b, and TNU20230511041d) clustered into a single branch with unnamed *Erythrotrichia* species from South Africa, Australia, America, and the Netherlands, receiving high support (ML bootstrap values = 86).

### ﻿Morphological results

#### ﻿ Erythrotrichia bohanensis

Taxon classificationPlantaeErythropeltidalesErythrotrichiaceae

Huang, Chu & Ding sp. nov.

39C1B793-7189-5257-AF5E-7AA81DA5D7F2

##### Description.

**Figure 3.**
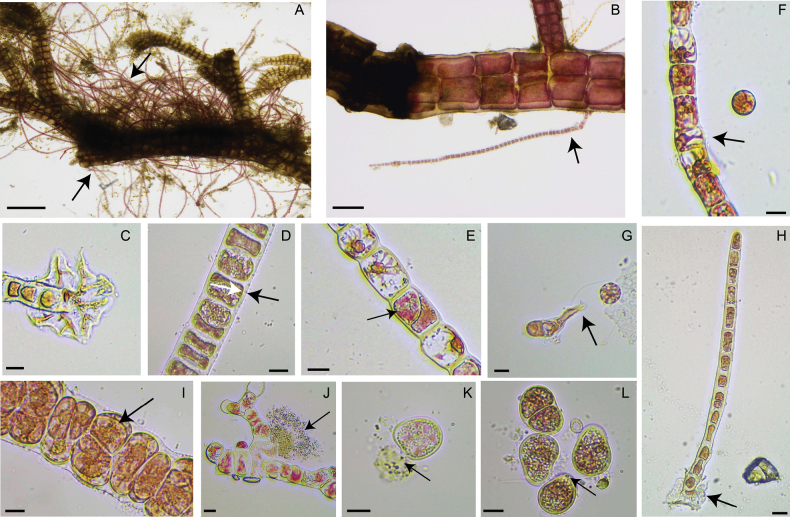
Microphotographs of *Erythrotrichiabohanensis* Huang, Chu *&* Ding, sp. nov. **A, B** epiphytic state on host alga *Polysiphonia* sp., arrowheads point to *E.bohanensis***C** base of the alga, arrowheads point to basal discoid cell **D** cells are enveloped by a mucilaginous membrane, arrowheads point to the gelatinous membrane **E** vegetative cell dividing to form a monosporangium, arrowheads point to a monosporangium **F–H** monospores sprouting to form seedling **I** vegetative cells expand and divide to form carpogonium, arrowheads point to carpogonium **J** male gametophyte releases sperms, arrowheads point to sperms **K** spermatium released sperms outside the alga, arrowheads point to sperms **L** fertilization and zygotospore divide outside the alga. Scale bars: 500 μm (**A**); 100 μm (**B**); 10 μm (**C–L**).

Thallus rose-red, green to purple in color, with erect, mostly unbranched filaments. The thallus consists of a uniseriate row of cells. Mature thalli reach a height of 4.7–5.2 mm. The base of the thallus is discoid in shape (Fig. 3C), that facilitates attachment to the host algal surface (Fig. 3A, B). The diameter of the attachment disc ranges from 41.0 to 60.8 μm (Fig. 3C, H). The thallus tapers from bottom to top, with the diameter 19.5–22.7 μm at the base, 17.2–18.10 μm in the middle, and 9.7–11.4 μm in the upper part. Vegetative cells are elongate-ovoid in shape, with basal cells 13.2–16.0 μm in height and 8.2–11.9 μm in width, central cells 13.9–20.0 μm in height and 12.3–13.3 μm in width, and distal cells 15.0–17.6 μm in height and 7.2–8.0 μm in width. Apical cells are suborbicular, with a diameter of 8.3–8.5 μm. Cells are enveloped by a gelatinous membrane that lies external to the cell wall, 1–4 μm in thick (Fig. 3D).

Asexual reproduction occurs through monospores. Monosporangia are formed by vegetative cells dividing obliquely or longitudinally (Fig. 3E), and one of these cells is transformed into a monosporangium releasing monospores, measuring 11.5–15 μm in diameter (Fig. 3F, G). The monospores germinate into sporelings (Fig. 3F–H). During monospore germination, the cell undergoes polar division, extending on one side to form a uniseriate filament and on the other side to form a unicellular disk (Fig. 3F)or a polar pseudoroot with an expanding adhesive rhizoid tip (Fig. 3G). In sexual reproduction, carpogonia are formed by the expansion and division of vegetative cells (Fig. 3I), 17.0–22.0 μm in diameter. Spermatangia are derived from vegetative cells, exhibiting a lighter pigmentation compared to the latter. Spermatia are released after maturation in the male gametophyte (Fig. 3J) or outside the thallus (Fig. 3K). Colorless spermatia contact and fuse with carpogonia outside the thallus. Following fertilization the zygotes germinate into sporelings (Fig. 3L).

##### Holotype.

TNU20230511041b, epiphyte on *Polysiphoniamorrowii* Harvey.

##### Type locality.

Dongshan Beach, Qinhuangdao, Hebei Province, China(39°54'N, 119°37'E).

##### Isotypes.

TNU20230318010a epiphyte on *Polysiphoniasenticulosa* Harvey, and TNU20230511041c, TNU20230511041d epiphyte on *Polysiphoniamorrowii* Harvey. All specimens were preserved in the algae Laboratory, Tianjin Normal University, Tianjin, China.

##### Etymology.

*Bohanensis*, the holotype was collected from the Bohai Sea.

##### DNA sequences.

To be uploaded into Genbank.

##### Distribution and habitat.

Qinhuangdao, Hebei Province, China. Japan, Australia, America, the Netherlands, and South Africa. Epiphytic life, on macroalgae.

## ﻿Discussion

In this study, *rbc*L and SSU fragments from four samples (TNU20230318010a, TNU20230511041b, TNU20230511041c, TNU20230511041d), collected from the Bohai coast, were found to cluster in a single lineage together with species from Japan, South Africa, Australia, and the Netherlands. This clustering is supported by high ML bootstrap values and BI posterior probabilities, as shown in Figs 1, 2. These samples correspond precisely to Lineage 5 reported by West et al. (2012), showing differences at the molecular level from previously reported *E.carnea*, *E.welwitschii*, and *E.johnawestii*.

Morphologically, the samples in this study exhibit clear differences in reproductive structures and subtle differences in trophic structures compared to similar species, including *E.carnea*, *E.longistipitata*, *E.welwitschii*, and *E.johnawestii* (Magne 1990; Zheng and Li 2009; West et al. 2012; Wen et al. 2023), seen in Table 1.

**Table 1.** Morphological comparison between *Erythrotrichiabohanensis* sp. nov. and similar species.

Character	*E.carnea*	*E.longistipitata*	*E.welwitschii*	*E.johnawestii*	*E.bohanensis* sp. nov.
**Reproductive structure**					
Type of reproduction	asexual and sexual reproduction	asexual reproduction	asexual reproduction	asexual reproduction	asexual and sexual reproduction
Diameter of monospores (μm)	5–10	9–11	12–15	10–11	11.5–15
Diameter of carpogonia (μm)	Similar to vegetative cells				17–22
Diameter of spermatangium (μm)	Similar to vegetative cells				21
Diameter of spermatia(μm)	5				2–3
**Vegetative structure**					
branched or unbranched	unbranched	unbranched	branched	unbranched	unbranched
Length of thallus (mm)	5–30	1–2	5–6	–	4.7–5.2
Width of cells (μm)	10–13	10–20	15–23	16–19	7.2–13.3
Length of cells (μm)	6.5–7	13–18	12–23	6–11	13.2–20
Diameter of basal thallus (μm)	8–10	–	–	–	19.5–22.7
Diameter of thallus (μm)	13–25	–	–	–	9.7–18.1
Thickness of gelatinous membrane (μm)	3–5	–	–	–	1–4
Reference	Magne 1990; Zheng and Li 2009	West et al. 2012	West et al. 2012	Wen et al. 2023	This study

Asexual reproduction in the new species produces monospores through oblique or longitudinal division of vegetative cells. The monospores are slightly larger in diameter compared to *E.carnea*, *E.longistipitata*, and *E.johnawestii* (Zheng and Li 2009; West et al. 2012; Wen et al. 2023). During germination indoors, the monospores develop into a lobed unicellular disk, which subsequently leads to the formation of an adhesive rhizoid tip (Figs 3 I, J). As the rhizoid elongates, its adhesive tip broadens, facilitating attachment to the substrate. These characteristics are consistent with the morphological features of species from Australia and the Netherlands in lineage 5 as previously reported by Zuccarello et al. (2011).

Sexual reproduction has only been reported in *Erythrotrichiacarnea* in the genus. In *E.carnea*, gametophytes of consist of three cells, with the apical cell dividing obliquely to form a spermatangium, spermatia are 5 μm in diameter (Magne 1990). Following spermatia release, the remaining apical cell of male gametes transforms into the carpogonium (Magne 1990). Fertilization occurs directly at the tip of the thallus without the release of carpogonia (Magne 1990). However, the sexual reproductive history is often incomplete, and ploidy changes have yet to be clearly established (Zuccarello et al. 2010). In contrast, for *E.bohanensis*, spermatia and carpogonia are formed through the expansion and division of vegetative cells located in the middle of the thallus. Spermatia release occurs after maturation within the the male gametophyte (Fig. 3J) or outside the thallus (Fig. 3K), with fertilization taking place externally. This approach may be associated with replication versus meiosis but it needs further research to elucidate this relationship.

In terms of vegetative structure, the new species in this study are mostly unbranched filaments with a thicker basal diameter that tapers towards the upper part, and more elongated cells. Comparisons show subtle differences in vegetative characteristics among these species, with field-collected individuals showing even more minute variations, necessitating precise measurements. The probability of collecting individuals with critical sexual reproductive structures in the field is lower, adding to the challenge of identifying species within this taxon. Moreover, the new species has morphological overlap with the previously reported *E.carnea*, further suggesting that there may be hidden species within the genus previously reported based on morphology. Cryptic species are those that share subtle morphological features but differ genetically from each other (Cheng et al. 2024). Furthermore, the current development of molecular biology techniques has led to the discovery of a large number of cryptic species, and hidden biodiversity has attracted increasing attention (Cahill et al. 2024). The existence of undescribed cryptic species presents significant challenges for conservation biology (Hending 2024). While these diminutive epiphytic species in this study are frequently overlooked, they constitute integral components of global biodiversity. Their population fluctuations may serve as valuable bioindicators of shifting environmental conditions or declining ecosystem health. To reveal cryptic species, the most important method is molecular biology analysis (Cheng et al. 2024). Additionally, new methods such as chromosome ploidy analysis, regional analysis, and machine learning have been applied (Heine et al. 2024; Huang et al. 2024; Olivares et al. 2024). Experimental taxonomy can be effectively applied to tiny individuals, such as the taxon in this study. Utilizing minimal sample sizes to cultivate populations under controlled laboratory conditions not only reveals genetic insights but also yields pure samples essential for molecular identification. Future taxonomic studies on this taxon should concentrate on morphological classification, detailed morphological characterization, and the integrated application of taxonomic methods, including systematic and experimental taxonomy. By compiling more detailed morphological, geographical distribution, molecular, and genetic data, we can address the challenges of species identification and uncover cryptic species diversity.

## Supplementary Material

XML Treatment for Erythrotrichia bohanensis

## ﻿Additional information

### Data availability

All of the data that support the findings of this study are available in the main text or Supplementary Information.

## Supplementary materials

Supplementary material 1 GenBank accession numbers for phylogenetic analysis Data type docx https://binary.pensoft.net/file/1341228This dataset is made available under the Open Database License (http://opendatacommons.org/licenses/odbl/1.0/). The Open Database License (ODbL) is a license agreement intended to allow users to freely share, modify, and use this Dataset while maintaining this same freedom for others, provided that the original source and author(s) are credited.Bingxin Huang, Yue Chu, Yuan Gao, Yanguang Zhong, Meina Chen, Chang Sun, Lanping Ding
