# Supplementary material for: ﻿A new species Erythrotrichiabohanensis sp. nov. (Erythropeltales, Rhodophyta) from the coast of China
Source: PhytoKeys. 2025 May 29;256:175–84. doi: 10.3897/phytokeys.256.145842 (PMC12142218; doi:10.3897/phytokeys.256.145842)
Supplement: Supplementary material 1 — GenBank accession numbers for phylogenetic analysis [file phytokeys-256-175_article-145842__-s001.docx]

**Supplement data**

**Table S1 GenBank accession numbers for phylogenetic analysis**

| Species | *rbc*L Accession No. | Location | Country Code | SSU Accession No. | Location | Country Code |
| --- | --- | --- | --- | --- | --- | --- |
| *Erythrotrichia carnea* | U04040 | Japan | JP | AY617155 | Netherlands | NL |
|  | AF087118 | America | US | AJ880417 | Germany | DE |
|  | EF660274 | Australia | AU | EF660312 | New Zealand | NZ |
|  | EF660275 | Australia | AU | JF292707 | Ireland | IE |
|  | EF660276 | Australia | AU | JF292708 | United Kingdom | GB |
|  | EF660277 | Madagascar | MG | JF292710 | United Kingdom | GB |
|  | JF292612 | United Kingdom | GB | JF292725 | Australia | AU |
|  | JF292614 | United Kingdom | GB | JF292739 | Japan | JP |
|  | JF292631 | Australia | AU | ON188796 | Spain | ES |
|  | OP432319 | Korea | KR | / | / | / |
| *Erythrotrichia johnawestii* | OP432318 | Korea | KR | / | / | / |
| *Erythrotrichia foliiformis* | / | / | / | GQ280849 | Australia | AU |
| *Erythrotrichia welwitschii* | JF292632 | France | FR | JF292726 | France | FR |
|  | JF292633 | France | FR | JF292742 | France | FR |
|  | JF292647 | France | FR | JF292706 | Australia | AU |
| *Erythrotrichia* sp. | JF292610 | Australia | AU | JF292711 | New Zealand | NZ |
|  | JF292615 | Australia | AU | JF292712 | New Zealand | NZ |
|  | JF292616 | Australia | AU | JF292713 | New Zealand | NZ |
|  | JF292617 | Australia | AU | JF292715 | Australia | AU |
|  | JF292618 | Australia | AU | JF292716 | Australia | AU |
|  | JF292619 | Australia | AU | JF292717 | Australia | AU |
|  | JF292620 | Australia | AU | JF292718 | Australia | AU |
|  | JF292621 | Australia | AU | JF292719 | Australia | AU |
|  | JF292622 | Australia | AU | JF292720 | America | US |
|  | JF292623 | America | US | JF292721 | New Zealand | NZ |
|  | JF292624 | New Caledonia | NC | JF292722 | New Caledonia | NC |
|  | JF292625 | Australia | AU | JF292723 | Australia | AU |
|  | JF292626 | Australia | AU | JF292724 | Australia | AU |
|  | JF292627 | Australia | AU | JF292728 | United Kingdom | GB |
|  | JF292628 | Australia | AU | JF292730 | Netherlands | NL |
|  | JF292629 | Australia | AU | JF292731 | France | FR |
|  | JF292630 | Australia | AU | JF292732 | Madagascar | MG |
|  | JF292634 | United Kingdom | GB | JF292733 | Madagascar | MG |
|  | JF292635 | France | FR | JF292738 | Brazil | BR |
|  | JF292636 | Netherlands | NL | JF292740 | Japan | JP |
|  | JF292637 | France | FR | JF292741 | Australia | AU |
|  | JF292638 | Madagascar | MG | JF292745 | United Kingdom | GB |
|  | JF292639 | South African | ZA | GQ280850 | Australia | AU |
|  | OP491550 | Kuwait | KW | GQ280851 | Australia | AU |
|  | / | / | / | GQ280852 | Australia | AU |
|  | / | / | / | GQ280853 | Australia | AU |
|  | / | / | / | GQ280854 | Australia | AU |
|  | / | / | / | GQ280855 | Australia | AU |
|  | / | / | / | GQ280856 | New Zealand | NZ |
|  | / | / | / | GQ280857 | Australia | AU |
| *Porphyrostromium japonicum* | JF292644 |  |  |  |  |  |
| *Porphyrostromium ligulatum* | JF292651 |  |  | JF292746 |  |  |
| *Rhodochaete pulchella* | AY119777 |  |  | AF139462 |  |  |
